# ApolipoproteinA1-75 G/A (M1^-^) polymorphism and Lipoprotein(a); Anti- vs. Pro-Atherogenic properties

**DOI:** 10.1186/1476-511X-6-19

**Published:** 2007-08-06

**Authors:** Ali I Albahrani, Jannete Usher J, Mohammed Alkindi, Eileen Marks, L Ranganath, Said Al-yahyaee

**Affiliations:** 1 Department of Clinical biochemistry, St Mary's Hospital, Newport, Isle of Wight, PO30 5TG, UK; 2 Department of Clinical biochemistry, Royal Liverpool University Hospital^1^, Duncan Building, 4^th ^floor, Liverpool, L69 3GA, UK; 3 Department of Clinical Biochemistry, Sultan Qaboos University Hospital, Muscat, PO box 123, Sultanate of Oman

## Abstract

**Background:**

ApolipoproteinA1(apoA1) is the major apoprotein constituent of high-density-lipoprotein(HDL). The relationship of apoA1 -75 bp(M1^-^) allele polymorphism with lipoprotein phenotype and cardiovascular diseae (CVD) remain unclear.

Overnight fasting blood samples were collected from a cohort of high-risk Omani population, 90 non-diabetic subjects and 149 type 2 diabetes mellitus (T2DM) subjects for genotype and phenotype studies.

**Results:**

The M1+ and M1- alleles frequencies were 0.808 and 0.192 for M1+ and M1-, respectively, comparable to the frequency of apoA1 (M1+ and M1-) amongst a healthy Omani population, 0.788 and 0.212, respectively.

The frequencies of the hetero- and homozygous subjects for the MspI polymorphism at -75 (M1^-^) of the apoA1 gene were in Hardy-Weinberg equilibrium.

The mean Lp(a) concentration was significantly higher(P = 0.02) in subjects carrying M1^- ^allele compared to M1^+ ^allele of the APOA1 gene with an odd ratio of 2.3(95% CI, 1.13–14.3), irrespective of gender and the diabetic status.

**Conclusion:**

ApolipoproteinA1-75 G/A (M1^-^) polymorphism is relatively common and is positively associated with Lp(a) and therefore, may confer a potential risk for cardiovascular disease (CVD).

## Background

Apolipoprotein-A1 (apoA1) is the major apoprotein constituent of HDL and an in vivo activator of lecithin-cholesterol-acyltransferrase (LCAT). An inverse relationship has been reported between the apoA1 and HDL and CVD in the general population [1,2]. The protected effect of apoA1 and HDL is mediated mainly through the promotion of cholesterol efflux from peripheral cells. In addition, both HDL and apoA1 may also have antioxidant, antithromobotic, and anti-inflammatory properties, which could have important anti-atherogenic effects [3]. The plasma concentrations of apoA1 and HDL are reported to be influenced by gender, BMI, age, and a number of environmental factors, e.g. smoking, alcohol intake, exercise and lipid lowering medications [1,3]. A strong genetic regulation of the concentration of HDL and apoA1 has been established in twin and family studies, the heritability of HDL concentration is around 40% [3,4]. A number of studies had reported that the inter-individual variations in plasma apoA1 and HDL are influenced to a great extent by a common polymorphism of a guanine (G) to adenine (A) substitution (G/A) at -75-bp (M1^-^) in the apoA1 promoter region of the apoA1 gene [5,6]. Numerous studies have looked into the above association with conflicting reports, part of the inconsistencies could be due to the interaction of various environmental and iatrogenic factors [4].

Previously, our group has reported the alleles frequency of apoA1 gene among a healthy Omani population. The frequency M1^- ^allele was 22% [7]. This study was undertaken to assess the influence of apoA1 -75 bp(M1^-^) allele polymorphism on lipoprotein phenotypes and Lp(a) among a cohort of high-risk of Omani diabetic and non-diabetic population.

## Results

Lp(a), TG, and HDLc were positively skewed, therefore, natural logarithms of the data were used in all parametric significance testing.

The frequencies of the hetero- and homozygous subjects for the MspI polymorphism at -75 (M1^-^) of the apoA1 gene were in Hardy-Weinberg equilibrium.

The M1+ and M1- alleles frequencies were 0.808 and 0.192 for M1+ and M1-, respectively. Table 1 represents the influence of apoA -75 bp (M1^-^) gene polymorphism on lipoprotein phenotype. The sample size of subjects carrying the homozygous minus alleles was small and therefore the hetero and homozygous minus alleles were combined together in one group. Neither the demographics nor most of the lipoprotein parameters other than Lp(a) have reached statistical significance. The mean Lp(a) concentration was significantly higher (P = 0.02) in subjects carrying the hetero or homozygous M1^-+/-- ^gene compared to M1^++ ^gene with an odd ratio of 2.3 (95% CI, 1.13–14.3).

**Table 1:** The demographic and lipoprotein phenotypes based on apoA1 genotypes.

	**ApoA-1 genotypes**		**P value**
	**M1**^++^	**M1**^+-/--^	
**Number**	151	84	
**age**	45.4(0.9)	45.2 (1.0)	**NS**
**BMI**	30.7(0.6)	31.9 (1.6)	**NS**
**%CVD**	28	27	**NS**
**%T2DM**	64	59	**NS**
**%smoker**	23	20	**NS**
**Lp(a) g/l**	0.200 (0.02)	0.285(0.03)	**0.02**
**ApoA-1 (g/l)**	1.19(0.02)	1.18(0.03)	**NS**
**HDLc mmol/l**	1.20(0.03)	1.21(0.04)	**NS**
**TChol mmol/l**	5.8 (0.12)	5.7(0.13)	**NS**
**LDLc (mmol/l)**	3.8 (0.11)	3.8(0.12)	**NS**
**ApoB (g/l)**	1.25(0.03)	1.25(0.03)	**NS**
**Triglycerides (mmol/l)**	1.9 (0.16)	1.7(0.11)	**NA**

Women have significantly higher TChol, LDLc apoB, HDLc, apoA1 and Lp(a) concentrations compared to men. Despite the confounding influence of gender on various lipids and lipoproteins, Lp(a) was significantly higher in subjects carrying the hetero or homozygous minus allele (M1^-+/-- ^gene) compared to the M1^++ ^gene, irrespective to gender (Table 2).

**Table 2:** The demographic and lipoprotein phenotypes based on gender and apoA1 genotypes.

	**Female** **ApoA-1 genotypes**			**Male** **ApoA-1 genotypes**		
	**M1**^++^	**M1**^+-/--^	**P value**	**M1**^++^	**M1**^+-/--^	**P value**
**Number**	56	30		95	54	
**age**	45.2 (1.2)	43.8(1.2)	**NS**	45.7(1.09)	45.9(1.2)	**NS**
**BMI**	29.6(4.5)	31(5.6)	**NS**	29.7(0.7)	31.2(2.2)	**NS**
**%IHD**	19	9	**NS**	34	38	**NS**
**%T2DM**	65	50	**NS**	63	65	**NS**
**Lp(a) g/l**	0.217(0.03)	0.368(0.07)	**0.03**	0.188(0.03)	0.247 (0.04)	**0.048**
**ApoA-1 (g/l)**	1.22(0.02)	1.26(0.04)	**NS**	1.16(0.02)	1.14(0.02)	**NS**
**HDLc mmol/l**	1.3 (0.05)	1.3(0.07)	**NS**	1.15(0.03)	1.14(0.04)	**NS**
**TChol mmol/l**	5.86(0.2)	5.98 (0.2)	**NS**	5.76(0.15)	5.65(0.16)	**NS**
**LDLc (mmol/l)**	3.9(0.2)	3.98 (0.2)	**NS**	3.79(0.14)	3.68(0.14)	**NS**
**ApoB (g/l)**	1.27(0.05)	1.27(0.06)	**NS**	1.25(0.35)	1.23(0.3)	**NS**
**Triglycerides (mmol/l)**	1.82(0.17)	1.50 (0.17)	**NS**	2.09(0.23)	1.8(0.14)	**NS**

Lp(a) concentration was significantly higher in subjects carrying the hetero or homozygous M1^-+/-- ^gene compared to the M1^++ ^gene among women irrespective of the diabetic status. However, among men, it has reached statistical significance among the non-diabetic subjects only, (Table 3).

**Table 3:** the influence of apoA1 genotypes on Lp(a) based on gender and a) diabetic (non-diabetic (T2DM^-^) and diabetic (T2DM^+^)) and b) CVD (without CVD (CVD^-^) and with established (CVD^+^)).

**a)**						
	**Female** **ApoA-1 genotypes Lp(a) g/l**			**Male** **ApoA-1 genotypes Lp(a) g/l**		
	**M1**^++^	**M1**^+-/--^	**P value**	**M1**^++^	**M1**^+-/--^	**P value**

**T2DM**^-^	0.260(0.027)	0.312(0.023)	**<0.05**	0.174(0.02)	0.328(0.03)	**<0.05**
**T2DM**^+^	0.190(0.018)	0.420(0.038)	**<0.05**	0.146(0.014)	0.149(0.09)	**NS**

**b)**						
	**Female** **ApoA-1 genotypes Lp(a) g/l**			**Male** **ApoA-1 genotypes Lp(a) g/l**		

	**M1**^++^	**M1**^+-/--^	**P value**	**M1**^++^	**M1**^+-/--^	**P value**

**CVD**^-^	0.198(0.02)	0.374(0.03)	**<0.05**	0.153(0.0.2)	0.246(0.04)	**<0.05**
**CVD**^+^	0.273(0.02)	0.320(0.01)	**NS**	0.218(0.02)	0.190(0.018)	**NS**

## Discussions

The G-to-A transition in the promoter region of apoA1 gene -75 (M1^-^) is relatively common, occurring in around 20% of the adult Omani diabetic and non-diabetic population. Quite comparable to what our group have reported previously on healthy Omani adult population.

The present study has revealed that the G-to-A transition (M1^-^) in the promoter region of APOA1 gene was associated with a significantly higher Lp(a) concentration in both diabetic and non-diabetic subjects irrespective of gender. We underwent a Medline search; we were only able to find one study where an association between the apoA1 alleles polymorphism and Lp(a) was reported. This study was on newborn babies from an Asian origin (Singapore, Chinese, Malays and Asian Indian) and on samples collected from cord plasma. They undertook this study free of the confounding effect of environmental factors to determine the influence of apoA1 gene -75 (M1^-^) polymorphism on apoA1 and HDL concentrations. To their surprise no significant association was revealed between the apoA1 polymorphism and the apoA1 and HDL, however, a significant association was found between the above polymorphic sites and Lp(a) in male Asian Indians, and the apoA1 gene could account for 14% of the variation in Lp(a) concentration [2].

The present study did not reveal any significant association or influence of apoA1 polymorphism on the lipoprotein phenotype other than Lp(a). Partly due to the fact that diabetes and metabolic syndrome are highly prevalent in Oman, according to a national survey, 20–30% of the adult Omani population are either T2DM or have metabolic syndrome partly due to the increase in prevalence of obesity, sedentary life and consanguinity rate. T2DM is associated with dyslipidaemia that is characterized by raised triglyceride, and low HDL cholesterol and therefore, it is tempting to speculate such confounding factors could mask the influence of apoA1 genotyping on lipoprotein phenotype [8-11]. Nevertheless, controversy exists in the literature with regard to the influence of apoA1 polymorphism on HDL cholesterol, LDL and apoB. A significant associations between the M1^- ^allele and elevated HDL-cholesterol or apo A-I concentrations were reported in Italian boys, but not girls [12]; in healthy, physically active boys and young males from Belgium [13]; in Finnish men [14] and in French-Canadian women [15]. In contrast, no associations of the M1^- ^allele with HDL-cholesterol or apo A-I concentrations were reported by Civeira et al [16], Lopez-Miranda et al [17], Mata et al [18], Carmena-Ramon et al [19], Akita et al [20] or Barre et al [21]. Moreover, Matsunaga et al in their study observed that control subjects with the *G/A *genotype had significantly lower plasma concentrations of apoA1 [24]. Pukkinen et al and Heng et al. in their study they did not report any association between the M1^- ^allele of the APOA1 gene and HDL/or apoA1 concentration in both diabetic and non-diabetic subejcts [23]. In contrast Meng et al., reported a lower concentration of HDLc and apoA1 among subjects carrying M1^- ^allele of the APOA1 gene [14].

Numerous studies have confirmed the pro- and anti-atherogenic properties, respectively, of Lp(a) and apoA1 [24-26]. However, very few studies have looked into the relations or impact of plasma concentration of apoA1 on Lp(a). Animal studies have shown that the presence of both transgenic apo(a) plus apoA1 results in animals protected against diet-induced atherosclerosis [27]. Chabra et al. reported an association between apoA1 -75-bp and IHD without any emphasis to the underlying cause, however, lower HDLc level was noticed in their studied group [22]. In contrast, Petrovic et al. their study did not reveal an association between ApoA1 polymorphism and IHD [23]. The present study is the first in an adult population that highlights the influence of apoA1 polymorphism on Lp(a) concentration and the potential pro-atherogenic role of M1^- ^allele of the APOA1 gene. Future studies are required on larger samples to confirm our findings and perhaps look into some of the molecular mechanism that could explain the interaction of apoA1 polymorphism and Lp(a).

## Conclusion

The G-to-A transition (M1^-^) in the promoter region of APOA1 gene was significantly associated with higher Lp(a) concentration in irrespective of gender and diabetic status.

### Subjects and methods

The institutional Ethics Committee approved the study and all subjects gave their informed consent prior to participating in this study. Ninety non-diabetic subjects (27 women and 63 men) and one hundred and forty nine diabetic subjects (53 women and 96 men) were recruited from cardiac/lipid and diabetic clinics, respectively. All patients had cardiovascular, respiratory, and central nervous systems examination. Blood pressure was measured to the nearest even digit using a sphygmomanometer with the subject in the sitting position after a 5–10 minutes rest and considered hypertensive if the blood pressure was equal or greater than 140/90 mmHg on two repeated occasions or by 24 BP monitoring. Those on anti-hypertensive therapy were also assumed to be hypertensive. Diagnosis of T2DM was based on clinical characteristics; prior history of use of oral hypoglycaemic agents, the presence of obesity, no history of ketosis, or strong family history of diabetes. World Health Organization criteria for diagnosis of T2DM either by an abnormal oral glucose tolerance test (OGTT), two abnormal fasting blood glucose (>7.0 mmol/L) or high HbA1_c _(>7%) were fulfilled by all patients with type 2 diabetes. The proportions of T2DM subjects on insulin, oral hypoglycaemic agents or dietary control were 3%, 57% and 40%, respectively. History of smoking, alcohol, exercise and prophylactic drugs (e.g. β-blockers, aspirin, diuretics and sulphonylurias) was collected. Patients were labeled CVD^+ ^if they had a previous myocardial infarction more than three months prior to entry to the study or having stable angina pectoris and/or they had a positive thallium stress test or coronary angiogram. Exclusion criteria included myocardial infarction within three months prior to entry to the study, uncontrolled thyroid disease (hypo or hyperthyroidism), macro-proteinuria (positive urine protein dip-stick x2), severe hepatic impairment or renal impairment (serum creatinine level >114 (μmol/L), and those on lipid modifying agents.

An overnight fasting blood samples were taken for measurement of total cholesterol (TChol), triglycerides (TG), HDL-cholesterol (HDLc), Lp(a) and apoA1 and apolipoprotein B (ApoB). Cholesterol and triglycerides were measured using timed endpoint enzymatic methods on the Synchron CX system (Beckman).

ApoA1, apoB and Lp(a) were measured using rate nephelometric immunochemistry assay by IMMAGE system (Beckman). Both the ApoB and ApoA-1 methods used have been standardized according to the International Federation of Clinical Chemistry. Reference material for ApoB and ApoA-1 are lot SP3-07 and lot SP1-01, respectively, which were approved by WHO [18]. Low density lipoprotein cholesterol (LDLc) was calculated using the Friedwald formula and was not calculated when TG level was >4.4 mmol/L.

Polymerase chain reaction (PCR) was performed with a modification of the Wang, Badenhop et al (1996) method. Approximately 100 ng of genomic DNA was amplified in a 25-μl reaction mixture containing 1.5 m*M *Mgd_2_, 200 μ*M *dNTPs, 10 pmol of each primer (forward primer: 5'-AGGGACAGAGCTGATCC-TTGAACTCTTAG-3'; reverse primer 5'TTAG-GGGACACCTACCCGTACAGGAAGAGCA-3'), AND 1 u Taq DNA polymerase (GibcoBRL). PCR conditions consisted of a 5-min initial denaturation at 94°C, followed by 35 cycles of denaturation at 94°C for 1 min, annealing at 60°C for 0.5 min, and extension at 72°C for 0.5 min, with a final extension at 72°C for 5 min. Fifteen microliters of the 434-bp PCR product containing here *MspI *restriction sites at -75, +37, and +83-bp of the APOA1 gene (Shoulders et al. 1983) was digested with 3 U of *MspI *(Gibco BRL) overnight. Accordingly, the digested PCR products gave 67-, 113-, 45-, and 209-bp fragments for the wild type and a combination of these fragments for the rare alleles. These fragments were analyzed on 8% polyacrylamide gel stained with ethidium bromide. The genotypes were determined by comparing the relative mobilitys of DNA fragments with reference to size molecular weight markers. The genotypes and alleles were assigned the names adopted by Wand, Liu et al (1996) [7].

### Statistical Analysis

Data was analyzed with the Statistical Package for the Social Sciences Software (SPSS version 10).

Descriptive analysis including the estimation of mean values and standard error of the mean (SEM) for continuous variables were calculated. Skewed parameters were logarithmically transformed and a parametric test was used.

The significance of differences for the means of plasma Tchol, LDLc, HDLc, Triglycerides, apoB, apoA1, and Lp(a) between genders and various groups were determined by the t test or ANOVA as appropriate.

Chi square tests were used for establishing the relationship between the lipid parameters and the apoA1 alleles. The non-parametric Mann-Whitney test was used when the underlying assumptions were not met.

ANOVA was performed to determine the effects of the apoA1 gene polymorphism on the various lipid traits in the different groups after adjustment for significant covariates. In order to verify whether age and sex were effective modifiers in the estimation of odds ratios, separate binary logistic regression models were fitted for each of the lipid profiles after adjusting for age and sex as covariates. To study the relationship between apoA1 polymorphism and IHD, two separate binary logistic regression models were considered. Once the probability model for IHD due to only apoA1 polymorphism was fitted and then the model was refitted including all the six lipid profiles, for examining whether they were potentially effect modifying variables or not. A p-value (two-tailed) of less than 0.05 was considered as statistically significant.

## Competing interests

We declare that there have been no competing or financial interests.

## Authors' contributions

AAB, is the major author of the above study, he had a substantial contribution to the conception, design, analysis as well as interpretation of the data. Jeannette Usher's main role was the statistical analysis of the data. EM and LR have contributed to the drafting, revising as well as critically scrutinizing the manuscript. SA and MK have contributed to the recruitment and analysis of chemistries. All authors read and approved the final manuscript.
